# Comparison of Outcomes Between Metabolic Dysfunction-Associated Fatty Liver Disease and Non-alcoholic Fatty Liver Disease: A Meta-Analysis

**DOI:** 10.7759/cureus.44413

**Published:** 2023-08-30

**Authors:** Ghazala S Virk, Jaahnavi Vajje, Nausheen K Virk, Raam Mannam, Wajeeh Rehman, Naglaa G Ghobriel, Irfan-ud-din Mian, Muhammad Usama

**Affiliations:** 1 Internal Medicine, Avalon University School of Medicine, Youngstown, USA; 2 Internal Medicine, Dr. Pinnamaneni Siddhartha Institute of Medical Sciences and Research Foundation, Vijayawada, IND; 3 Internal Medicine, Ross University School of Medicine, Miramar, USA; 4 General Surgery, Narayana Medical College, Nellore, IND; 5 Internal Medicine, United Health Services Hospitals, Johnson City, USA; 6 Internal Medicine, State University of New York Upstate Medical University, Binghamton, USA; 7 Internal Medicine, Alexandria University, Alexandria, EGY; 8 Medicine, Combined Military Hospital (CMH) Lahore Medical College and Institute of Dentistry, Lahore, PAK; 9 Neurology, Sheikh Zayed Medical College and Hospital, Rahim Yar Khan, PAK

**Keywords:** systematic review and meta-analysis, cardiovascular outcomes, mortality, nonalcoholic fatty liver disease (nafld), metabolic-associated fatty liver disease (mafld)

## Abstract

Non-alcoholic fatty liver disease (NAFLD) encompasses a range of conditions, from fatty liver to cirrhosis. In response to evolving research and to better reflect the complex metabolic underpinnings, the term metabolic dysfunction-associated fatty liver disease (MAFLD) has been proposed. The aim of this meta-analysis was to compare cardiovascular events and all-cause mortality between NAFLD and MAFLD patients. The present study was conducted following the Preferred Reporting of Systematic Review and Meta-analysis (PRISMA) guidelines. We systematically searched PubMed, EMBASE, and the Web of Science to identify studies that compared cardiovascular outcomes in MAFLD and NAFLD from inception to July 31, 2023. Outcomes assessed in this meta-analysis included all-cause mortality, cardiovascular mortality, and cardiovascular events. A total of 11 studies were included in this meta-analysis. The risk of cardiovascular mortality was significantly higher in patients with MAFLD patients compared to NAFLD patients (risk ratio (RR): 1.48, 95% confidence interval (CI): 1.11 to 1.98). The risk of all-cause mortality was higher in MAFLD patients compared to NAFLD, and the difference was statistically significant (RR: 2.80, 95% CI: 2.39 to 3.28). The risk of cardiovascular events was significantly higher in MAFLD patients compared to NAFLD (RR: 1.18, 95% CI: 0.86 to 1.61). The key findings underscore that individuals diagnosed with MAFLD face a notably higher risk of all-cause mortality, cardiovascular mortality, and cardiovascular events when compared to those with NAFLD.

## Introduction and background

Non-alcoholic fatty liver disease (NAFLD) encompasses a range of conditions, from fatty liver to cirrhosis [1]. The diagnosis of NAFLD involves ruling out secondary causes of liver fat accumulation, like excessive alcohol consumption, use of fatty liver-inducing medications, viral hepatitis, and autoimmune liver ailments [1]. Recently, criticisms have emerged about the NAFLD nomenclature for not adequately considering the combined effects of these factors and the role of metabolic variables [2]. To address this, a panel of experts from the European Liver Patients' Association proposed a more fitting term in 2020: metabolic dysfunction-associated fatty liver disease (MAFLD), aimed at describing liver disease resulting from metabolic dysfunction [3]. The proposed MAFLD diagnostic criteria involve the presence of liver fat accumulation alongside obesity, diabetes mellitus, or metabolic dysfunction markers without requiring a specific underlying cause.

MAFLD stands apart from prior NAFLD diagnostic criteria [4]. The two major distinctions are that MAFLD diagnosis doesn't involve excluding patients with alcohol use or other chronic liver conditions [5], and it necessitates the presence of metabolic irregularities [6]. Emerging evidence suggests that MAFLD provides better indications of severe clinical outcomes compared to NAFLD [7]. In a Japanese study of 765 individuals with fatty liver, those with MAFLD displayed higher liver stiffness as measured by elastography (7.7 kPa vs. 6.8 kPa) and greater sensitivity in detecting significant fibrosis (93.9% vs. 73%) compared to NAFLD [8]. Similarly, an analysis of the Third National Health and Nutrition Examination Survey (NHANES III) indicated that MAFLD was superior to NAFLD in identifying individuals at high risk for advanced fibrosis [9].

In NAFLD patients, cardiovascular disease (CVD) is the primary cause of death [10,11], while only a minority experience severe liver disease or liver-related mortality [12]. Hence, CVD prevention is crucial in MAFLD management. The revised diagnostic criteria for MAFLD are more closely linked with an elevated CVD risk, given the associations between MAFLD diagnosis and established CVD risk factors like abdominal obesity, hypertension, atherogenic dyslipidemia, and insulin resistance/dysglycemia [13]. However, the extent of these associations and their impact on MAFLD development are not fully understood. Given the metabolic focus of the new MAFLD definition and the potential coexistence of other liver conditions with hepatic steatosis, it is reasonable to anticipate an increased risk of cardiovascular complications. However, comprehensive and conclusive data on cardiovascular changes in MAFLD are scarce, likely due to the recent introduction of the MAFLD concept. As a result, we are conducting a meta-analysis using available studies to compare cardiovascular events and all-cause mortality between NAFLD and MAFLD patients.

## Review

Methodology

The present study was conducted following the Preferred Reporting of Systematic Review and Meta-analysis (PRISMA) guidelines. We systematically searched PubMed, EMBASE, and the Web of Science to identify studies that compared cardiovascular outcomes in MAFLD and NAFLD from inception to July 31, 2023. In Embase and PubMed, we combined medical subject heading terms, text words, and truncation when relevant. This method involved a combination of the illness (MAFLD), the comparison group (NAFLD), and the aforementioned filter. To further refine the search, we also included an outcome in our search strategy. We manually screened the reference lists of the included studies to identify potential additional studies.

Selection Criteria and Data Abstraction

We included studies (both prospective and retrospective) that compared NAFLD and MAFLD and reported at least one relevant outcome. No exclusion was based on follow-up duration or sample size. We excluded studies that lacked a comparison group and those published in languages other than English. Two independent reviewers screened all records obtained through database searching. After removing duplicates, the initial records were screened based on the aforementioned inclusion and exclusion criteria. Full texts were obtained for eligible studies, and a detailed assessment was performed. Data from included studies were collected using a pre-designed data abstraction form, which included details such as author name, publication year, study arms, sample size, follow-up duration, baseline characteristics, and outcomes. Outcomes assessed in this meta-analysis included all-cause mortality, cardiovascular mortality, and cardiovascular events.

Quality Assessment

The quality of the included studies was assessed using the Newcastle-Ottawa Scale (NOS). The NOS assigns stars or points to various components of the study, evaluating three key domains: selection of study groups, comparability of groups, and assessment of outcomes. The total number of stars awarded reflects the overall study quality.

Statistical Analysis

We conducted a meta-analysis by pooling the data using either a random-effects or fixed-effects model with the Mantel-Haenszel method. Risk ratios (RR) with 95% confidence intervals (CI) were reported for all outcomes, and a significance level was set at a two-sided alpha of less than 0.05. Heterogeneity was assessed using the I-square statistic. An I-square value of more than 50% was considered significant for heterogeneity. To compare baseline characteristics between NAFLD and MAFLD, we calculated odds ratios (OR) with 95% CI, and for continuous outcomes, we reported the mean difference with 95% CI.

Results

**Figure 1:**
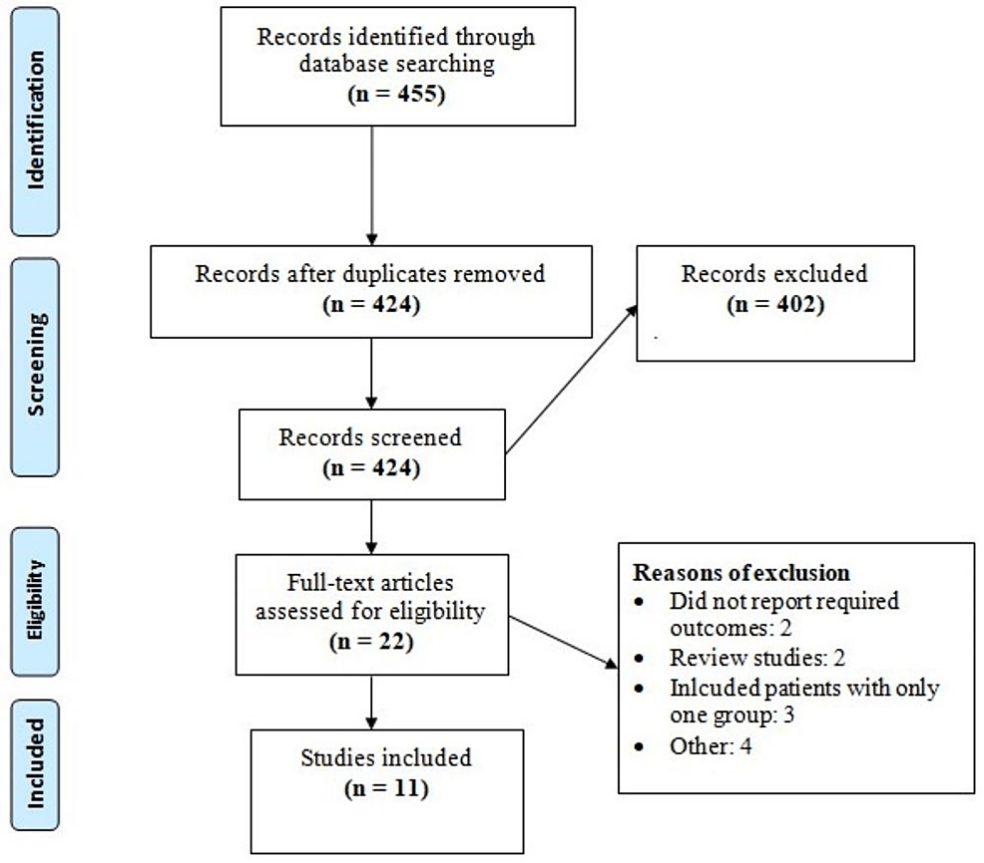
PRISMA flowchart of study selection. PRISMA: Preferred Reporting of Systematic Review and Meta-analysis.

Electronic databases and manual searches yielded 455 studies. After removing duplicates, we initially screened the titles and abstracts of 424 studies. The full texts of 22 studies were obtained, and on the basis of a detailed assessment, 11 studies were included in this meta-analysis. Figure 1 shows the process of study selection. Table 1 shows the characteristics of the included studies. The follow-up of the included studies ranged from 5.2 to 23.2 years. Table 2 shows the quality assessment of the included studies. Table 3 shows the characteristics of the included studies. As shown in Table 3, the odds of diabetes, hypertension, and dyslipidemia were significantly higher in MAFLD compared to NAFLD. Moreover, patients with MAFLD were significantly older compared to NAFLD. However, no significant differences were reported between the two groups in terms of body mass index (BMI).

**Table 1:** Characteristics of included studies. MAFLD: metabolic dysfunction-associated fatty liver disease; NAFLD: non-alcoholic fatty liver disease; NR: not reported.

Author name	Year	Region	Groups	Sample size	Follow-up
Guerreiro et al. [14]	2021	Brazil	MAFLD	154	NR
			NAFLD	109	
Huang et al. [15]	2021	China	MAFLD	658	22.8 Years
			NAFLD	528	
Kim et al. [16]	2021	United States	MAFLD	2256	23.2 Years
			NAFLD	2438	
Kim et al. [17]	2023	United States	MAFLD	16952	5.7 Years
			NAFLD	5979	
Lee et al. [18]	2021	Korea	MAFLD	948323	10.1 Years
			NAFLD	54896	
Nguyen et al. [19]	2021	United States	MAFLD	503	15 Years
			NAFLD	254	
Niriella et al. [20]	2020	Italy	MAFLD	735	7 Years
			NAFLD	708	
Semmler et al. [21]	2021	Austria	MAFLD	221	7.5 Years
			NAFLD	73	
Xu et al. [22]	2023	China	MAFLD	22835	5.2 Years
			NAFLD	20507	
Yoo et al. [23]	2023	United States	MAFLD	177731	8.8 Years
			NAFLD	157548	
Zhang et al. [24]	2021	China	MAFLD	7131	10 Years
			NAFLD	6658	

**Table 2:** Quality assessment of included studies.

Author name	Selection	Exposure	Outcome	Overall
Guerreiro et al. [14]	***	**	**	Good
Huang et al. [15]	***	*	**	Good
Kim et al. [16]	**	*	**	Fair
Kim et al. [17]	***	**	**	Good
Lee et al. [18]	***	*	***	Good
Nguyen et al. [19]	***	**	**	Good
Niriella et al. [20]	**	**	**	Fair
Semmler et al. [21]	***	**	***	Good
Xu et al. [22]	***	**	**	Good
Yoo et al. [23]	**	**	***	Good
Zhang et al. [24]	***	*	***	Good

**Table 3:** Comparison of baseline characteristics between MAFLD and NAFLD. OR: odds ratio; CI: confidence interval; BMI: body mass index; MAFLD: metabolic dysfunction-associated fatty liver disease; NAFLD: non-alcoholic fatty liver disease. *Reported as mean difference.

Variable	OR	95% CI	P-value
Age*	2.31	0.61, 4.01	0.008
Male	1.14	1.08, 1.20	0.001
Diabetes	7.93	2.59, 24.26	0.001
Hypertension	3.33	1.45, 7.65	0.005
BMI*	4.65	–0.36, 9.66	0.07
Dyslipidemia	2.03	1.35, 3.06	0.001

Cardiovascular Mortality

**Figure 2:**
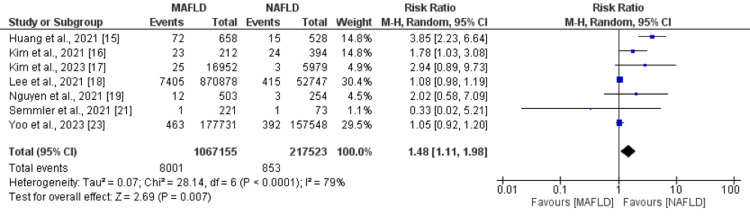
Cardiovascular mortality. MAFLD: metabolic dysfunction-associated fatty liver disease; NAFLD: non-alcoholic fatty liver disease. Sources: References [15-19,21,23].

We included seven studies in the pooled analysis of cardiovascular mortality. The risk of cardiovascular mortality was significantly higher in patients with MAFLD patients compared to NAFLD patients (RR: 1.48, 95% CI: 1.11 to 1.98), as shown in Figure 2. Significant heterogeneity was reported among the study results (I-square: 79%).

All-Cause Mortality

**Figure 3:**
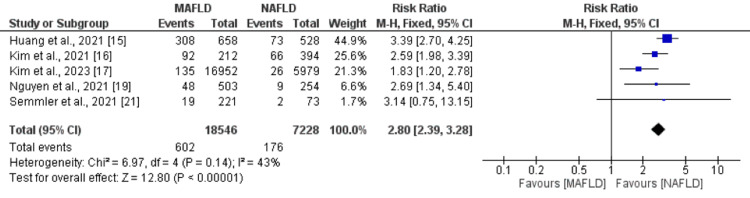
All-cause mortality. MAFLD: metabolic dysfunction-associated fatty liver disease; NAFLD: non-alcoholic fatty liver disease. Sources: References [15-17,19,21].

Five studies compared the risk of all-cause mortality between MAFLD and NAFLD patients. The risk of all-cause mortality was higher in MAFLD patients compared to NAFLD, and the difference was statistically significant (RR: 2.80, 95% CI: 2.39 to 3.28), as shown in Figure 3. No significant heterogeneity was reported among the study results (I-square: 43%).

Cardiovascular Events

Three studies were included in the pooled analysis of cardiovascular events. As shown in Table 4, the risk of cardiovascular events was significantly higher in MAFLD patients compared to NAFLD (RR: 1.18, 95% CI: 0.86 to 1.61). No significant heterogeneity was reported among the study results (I-square: 0%). We analyzed myocardial infarction and stroke separately. However, the risk of both events was higher in MAFLD patients compared to NAFLD, but the differences were statistically insignificant.

**Table 4:** Cardiovascular events. RR: risk ratio; CI: confidence interval.

Outcomes	No. of studies	RR (95% CI)	I-square
Cardiovascular events	3	1.31 (1.24 to 1.39)	0%
Myocardial infarction	3	1.05 (0.97 to 1.14)	20%
Stroke	2	1.18 (0.86 to 1.61)	95%

Discussion

This is the first meta-analysis, to our knowledge, that compares cardiovascular outcomes in patients diagnosed with NAFLD and MAFLD criteria. The key findings of this meta-analysis reveal that, when compared to NAFLD, patients diagnosed with MAFLD face a higher risk of all-cause mortality, cardiovascular mortality, and cardiovascular events. Our study also highlights that MAFLD patients, in comparison with NAFLD, were notably older and exhibited higher proportions of hypertension, diabetes, and dyslipidemia.

MAFLD is characterized by a stronger emphasis on metabolic factors, encompassing insulin resistance, obesity, dyslipidemia, and diabetes, which are well-established contributors to cardiovascular diseases [25]. Studies suggest that MAFLD patients often bear a heavier burden of metabolic risk factors compared to NAFLD patients. Consequently, the increased prevalence of metabolic dysfunction in MAFLD may lead to a heightened risk of cardiovascular events and related mortality compared to NAFLD participants [26,27]. Furthermore, MAFLD is increasingly recognized as a condition with systemic effects extending beyond the liver. Chronic inflammation and adipose tissue dysfunction, alongside interactions with other organs like the gut, contribute to the complexity of MAFLD [28]. This systemic inflammation can contribute to a heightened risk not only of cardiovascular events but also of other complications and mortality. In contrast, NAFLD, while also associated with inflammation, might exhibit a relatively milder systemic impact [29].

Since a consortium of global experts introduced MAFLD as a replacement for NAFLD, extensive discussions and concerns have arisen regarding this new term. However, over time, the nomenclature of MAFLD has gained more approval and support [30,31]. Since the release of the MAFLD definition, scientists and clinicians have been engaged in discussions to redefine the nosological framework for NAFLD [32]. The recognition of MAFLD, along with its emphasis on metabolic factors, is relatively recent, and our understanding of its implications continues to evolve. Previous studies might not have fully captured the distinct characteristics and risks associated with MAFLD. As further research is conducted and our understanding deepens, the observed differences in mortality risk between MAFLD and NAFLD may become more nuanced.

Although there is substantial overlap between MAFLD and conventional cardiovascular disease (CVD) risk factors, recent research suggests that MAFLD can independently predict negative CVD outcomes beyond these traditional factors [27]. Current evidence increasingly supports the link between MAFLD and CVD, which hepatologists widely acknowledge [33,34]. However, the role of MAFLD as a novel CVD risk factor is not yet fully recognized and often remains undiagnosed, unlike several other established CVD risk factors [27]. Given that a significant majority of global deaths now stem from chronic, lifestyle-related conditions (such as obesity, hypertension, and diabetes), interdisciplinary cooperation is imperative to enhance patient well-being [35]. In this context, raising awareness about the adverse metabolic and cardiovascular effects of MAFLD within the cardiology community could contribute to alleviating the worldwide burden of chronic, lifestyle-related diseases.

The study possesses certain limitations. Firstly, liver fibrosis was not assessed in the majority of the studies, and considering the significance of liver fibrosis in mortality, future studies should investigate its role to comprehend its impact on MAFLD and NAFLD. Secondly, cardiovascular events were not evaluated in most of the included studies. Thirdly, due to the absence of individual-level data, we were unable to perform subgroup analysis, which could provide more insights into the risk of adverse events across various patient groups.

## Conclusions

In conclusion, this meta-analysis represents a pioneering effort in comparing cardiovascular outcomes between patients diagnosed with NAFLD and MAFLD criteria. The key findings underscore that individuals diagnosed with MAFLD face a notably higher risk of all-cause mortality, cardiovascular mortality, and cardiovascular events when compared to those with NAFLD. The evolving recognition of MAFLD and its impact on cardiovascular health prompts us to consider a more holistic approach to patient management. As the understanding of MAFLD deepens, further research is warranted to unravel the intricate pathophysiological mechanisms that underlie its association with adverse cardiovascular outcomes. Additionally, future investigations should delve into the nuanced interplay between MAFLD, liver fibrosis, and cardiovascular risk to provide a more comprehensive understanding of these complex relationships.
